# The impact of medical teleconsultations on general practitioner-patient communication during COVID- 19: A case study from Poland

**DOI:** 10.1371/journal.pone.0254960

**Published:** 2021-07-16

**Authors:** Magdalena Kludacz-Alessandri, Liliana Hawrysz, Piotr Korneta, Grażyna Gierszewska, Wioletta Pomaranik, Renata Walczak

**Affiliations:** Warsaw University of Technology, Warsaw, Poland; Universita degli Studi della Campania Luigi Vanvitelli, ITALY

## Abstract

According to the outbreak of the Covid-19 pandemic, medical teleconsultations using various technologies have become an important tool to mediate communication between general practitioners (GP) and the patients in primary health care in many countries. The quality of the GP-patient communication is an essential factor, which improves the results of treatment and patient satisfaction. The objective of this paper is to study patients’ satisfaction from teleconsultation in primary care and the impact of teleconsultations on GP-patient communication through the Covid-19 pandemic in Poland. We analyse whether the teleconsultations performed without physical examinations have a positive impact on GP-patient communication. The quality of teleconsultation and GP-patient communication have been measured using a questionnaire regarding the quality of medical care in a remote care conditions. Among 36 items, nine questions have been related to the dimension of GP-patient communication and ten to system experience. Our results suggest that the quality of teleconsultations is not inferior to the quality of consultation during a face-to-face visit. The patients indicated a high level of satisfaction regarding communication with their GP during teleconsultation. We have also identified that the technical quality and the sense of comfort during teleconsultation positively impact the communication quality.

## Introduction

The onset of the Covid-19 pandemic has significantly contributed to an extremely rapid transformation of healthcare systems worldwide, with telemedicine being one of its main drivers. This is due to the fact that in a pandemic situation, telemedicine is often the only possible form of patients care. Until March 2020, telemedicine was used globally on a very small scale. In the United States in 2019, 8% of all medical visits were teleconsultations [1]. Among the basic reasons indicated for the low use of telemedicine, the lack of comfort in using telemedicine technologies by patients and suppliers was primarily. This lack of patients comforts resulted mainly from the two major sources [2, 3]. The first one is a consequence of concern that telemedicine cannot convey the "humanity" required to support effective interactions with the patient [2, 4–6]. The second one, closely related to the first, is referred to in the literature as the system experience. In particular, it is associated with the ease, simplicity and efficiency of its use [2, 7]. The system experience is one of the most frequently measured dimensions of patient satisfaction from teleconsultations [8]. The researchers postulate the two major aspects of to the system experience: a physical and a behavioral ones. The discussion in the literature of the physical aspects of to the system experience is mostly focused on technical and operational support for patients during the teleconsultations. The awareness of patients that they can count on support in the event of any disruptions or technical problems was largely emphasized in many studies. Furthermore, this awareness was indicated as the most important factor influencing the perception of the quality of the teleconsultation [2, 9, 10]. Technical support provision is important for reducing the risk of increasing disproportions in access to healthcare [11] and ensuring the sustainability of the telemedicine use [3, 12]. Discussion on the behavioral aspects of to the system experience was mostly focused on the patient’s comfort during the teleconsultations [4, 5, 9]. Another researchers focused on to the patient’s comfort as a general phenomenon [9] with some of them meticulously analyzed individual aspects of the teleconsultation, paying attention, for example, to the elimination of noise from the office during the teleconsultation, emotional support, careful selection of appropriate words expressing empathy [2, 6]. From the point of view of the patient’s comfort, very important factors emphasized in the literature were time and cost savings resulting from the lack of the need to travel to the GP [7, 12, 13]. In the context of the behavioral aspect, the patients’ fears of their security violation and confidentiality disclosures on their personal health status were often underlined [9, 10]. A very important element of the patient’s comfort was a properly built relationship with the GP [6, 14]. One of the key elements of this relationship between the patient and GP, that can be assessed by the patient is the communication. This relates to partnership, respect and sharing of knowledge and information between patient and GP. For patients, particularly important are: the GP’s ability to listen to the patient, understanding his concerns, explanation and understanding health problems and treatment possibilities, respect, kindness, sharing of knowledge and information between GP and the patient. The scholars propose a number of dimensions to measure GP-patient communication, e.g. physician’s use of Patient-Centered Communication, Physician’s Clinical Competence and Skills, Physician’s Interpersonal Skills, Convenience of Visit [15]. However, in our opinion, the two dimensions play a critical roles: the interpersonal communication and the respect [16]. According to another researchers communication and respect for patients show the extent of accommodation to the patient’s needs and preferences. These refers to interpersonal quality of medical care [17]. Interpersonal communication focuses on ease of exchange information between the patient and the GP [18]. These depend on the GP’s ability to express and understand the patient’s concerns, explain health issues, and engage in collaborative decision. The variables regarding interpersonal communication should measure the accuracy of the information provided, the ability to listen and ask questions [16, 17, 19–21]. Respect, on the other hand, is the degree to which GP and support staff meet patients’ expectations of interpersonal treatment, show respect for their dignity, and provide adequate privacy [22]. The variables regarding respect should measure a time spent with the patient, patience and concern as well as courtesy and prudence [16, 17, 19–21]. According to some researchers, this aspect of the GP-patient relationship should also apply to the patient’s privacy [20, 23] and a trust to the GP [15, 20, 23, 24]. The trust of patients in their GPs is especially recognised as an essential feature of a therapeutic relationship and is related to increased patient satisfaction form quality of the GP-patient relationship [24, 25]. Little, however, is known what aspects of the relationship are associated with increased levels of trust. There is evidence that measures of the quality of the GP-patient relationship, including GP-interpersonal communication and respect, are associated with patient trust [25]. There is, however, contradictory evidence that trust is associated with the duration of the GP-patient relationship [26, 27]. Thus, in this study, trust was not considered in the dimension of communication.

The Covid-19 pandemic has increased the use of telemedicine, mostly for urgent and non-surgical visits. According to research conducted in the United States, only in the period from March 2 to April 14, 2020, the number of teleconsultations increased by 683% [1]. Researchers agree that the Covid-19 pandemic has made telemedicine inseparable from the provision of medical services. The key advantage of telemedicine over physical visits is to prevent both the patients and the doctors from the risk of the virus infection [13, 28, 29]. Researchers believe that telemedicine will not completely eliminate face-to-face (F2F) visits, but it can successfully replace a significant fraction of them [7, 11, 12, 29–31]. Given above, the in-depth research focused on the system experience comprising technical and behavioural aspects of telemedicine through the pandemic period appears highly recommended. The system experience is assumed to be the main factor affecting telemedicine patients satisfaction. The objective of this paper is to study patients’ satisfaction from teleconsultation in the primary care and the impact of teleconsultations on the quality of GP-patient communication through the Covid-19 pandemic in Poland. We aim to determine if remote teleconsultation’s technical and behavioural aspects correlate with patient satisfaction in the dimension of GP-patient communication. By teleconsultations, we primarily understand telemedicine in the form of phone calls, video calls using the communicator (WhatsApp, Skype) and video calls via the platforms (Teams, Zoom). We recognize that our understanding of telemedicine is simplified. Telemedicine is not only teleconsultation. In recent years, several digital tools for remote patient management have been developed to reduce the risk of infection during this pandemic [32–35]. Still, they have not been discussed in this article.

The paper is organised as follows. In the first section, we review the literature focusing on the two dimensions: system experience and communication of patients with GPs. In the second section, a research methodology is being provided. In the following section, we provide results concerning the quality of teleconsultations and patient satisfaction from communication with GPs during the Covid-19 pandemic in Poland. We also comment on the impact of the system experience and other determinants connected with characteristics of teleconsultations on GP-patient communication. The paper ends with conclusions and practical implications.

## Research methodology

The survey instrument was constructed based on the literature review. Literature-driven questions have been slightly changed for this study. Given the constraints associated with the lockdown during Covid-19, we approached four local primary care practices in Poland that had the infrastructure to conduct call interviews (call centres). The questionnaire was assessed from an ethical perspective by the Warsaw University of Technology Senate Committee for Professional Ethics. Also, oral consent has been obtained from the Management Board of Primary Health Care providers in Poland responsible for call centers. The interviews were held with patients who consented to participate in the study by phone. Each patient could withdraw from the study at any time or not answer all the questions.

The total number of patients registered in the practices was 46 700 patients (MD patients, paediatric patients, nurses, midwife patients). One clinic was located in a large country town (Radom), and the reaming three in the capital of Poland (Warsaw). We were informed by the representatives of aforementioned clinics, that collectively, in a period between Nov 2020 and Jan 2021 (i.e. 3 full months) the clinics provided 15,499 services, of which 9,034 were face to face visits and 6,465 teleconsultations (42% of all services). Since our interviewees possessed no detailed information on spilt of teleconsultations we asked them to approximate it. As a result we were informed that approximately 95% of teleconsultations were conducted by phone, while remaining 5% with other means of communications, as was confirmed in our study.

We have selected randomly from the database the patients over 18 years old who used a teleconsultation after 1st of November 2020 (the time when the clinic started providing medical care in this form), i.e. who used teleconsultation during the lockdown caused by the Covid -19 pandemic. All patients who agreed to answer the questions from the questionnaire were eligible and they were surveyed using the computer-assisted telephone interviewing (CATI) method. The interviews have been conducted during four days in February and March 2021, and it took an average of 20 minutes to complete one survey. The interviews have been conducted among 105 patients. The ethics committee required that no respondent should be obliged to answer the questions. This meant that the questions might not have been answered, or the respondents could quit the study without giving a reason. The aim of the study has been outlined in the introduction to the interview. Most of the patients agreed to the interview, but 6 of them wanted to finish the interview earlier, and finally, their answers have not been taken into account. Ninety-nine complete records were included in the study, which represents a response rate of 94%. Because the minimum acceptable ratio of observations to variables (5:1) has been fulfilled we have decided to stop this pilot survey. It is worth adding that some researchers accept the ratio of 3:1. The absolute minimum number of observations in the factor model is 50 [36].

The questionnaire measure patient satisfaction from remote care has been developed based on previous research instruments with minor modifications to make them applicable to telemedicine in Poland. Before the interviews, the questionnaire has been informally analysed by external experts to confirm that the selected variables are well suited to the selected dimensions of the quality of primary care. During the interview, respondents were asked to indicate to what extent they agreed with each of the statements using a 5-point Likert scale (where 1 = very strongly disagree and 5 = very strongly agree). One of the dimensions studied was GP-patient communication. Variables to measure communication between physician and patient dealt with two subdimensions: "interpersonal communication" and "respect". Similar variables and subdimensions have also been used in The General Practice Assessment Survey (GPAS) [16]. To measure interpersonal skills, we have asked questions about the accuracy of the information provided, the ability to listen and ask questions. To measure respect, we have used questions about the time spent with the patient, patience, empathy and care. The variables relating to interpersonal communication and respect are presented in Table 1.

**Table 1:** Variables used to measure the quality of communication with GP.

Potential factors	Variables
Interpersonal communication	The GP listened carefully to what I had to say
	The GP understandably provided me with information about further diagnostic and treatment procedures
	The GP gave comprehensive and clear answers to my questions
Respect	The medical consultation time was sufficient
	The GP was patient and attentive
	The GP treated me with kindness and respect
	My GP supported me and understood my emotional needs
	I believe the GP really cared about me and my health problems
	I trust my GP and can give him even very personal information

The variables that measured the experience with the system dealt with two subdimensions: "technical quality" and "behavioural quality". Similar variables were also used in the study Layfield *et al*., 2020 [7]. To measure the technical quality, we asked questions about the quality of the phone call and technical support received during the teleconsultation. To measure the behavioural quality, we used questions about the feeling of comfort, security and general satisfaction from the consultation. Ten proposed variables regarding technical and behavioural quality used to measure the system are presented in Table 2.

**Table 2:** Variables used to measure the system experience.

Potential factors	Variables
Technical quality	The teleconsultation went smoothly, without the technical problems
	I believe that the technical support I received during the teleconsultation was adequate
	I can easily use electronic medical records
Behavioural quality	I felt comfortable discussing my health problems with my GP over the phone or on the Internet
	I believe that teleconsultation helps to ensure the security and confidentiality of my data as a patient
	I believe that teleconsultation is an acceptable way to solve my health problem
	I believe that teleconsultation saves time
	I believe that teleconsultation saves cost
	I believe that the medical care I received during teleconsultation is as good as seeing my GP F2F
	I believe that teleconsultation is an acceptable way to solve my health problem

To prepare the final dimensions and variables for analysis, confirmatory factor analysis was performed. The main goal of this technique is to quantify the extent to which the correlations between several observed variables can be explained by several latent variables, which we call factors in our study. In this sense, the procedure reduces the number of variables, replacing them with fewer factors treated as constructs or latent variables.

The analysis regarding communication and system experience has been performed several times, assuming 2–4 factors. Finally, we identified a 2-factor solution as the best approximation for the data. The scores on 8 of the communication statements were combined to produce a single score reflecting how much patients valued communication with GP during teleconsultations. One variable (“The medical consultation time was sufficient”) regarding communication and four variables relating to the system experience has been removed for the final factors because of low factor loadings or lack of fit to the model. The results of the factor analysis are presented in Table 3.

**Table 3:** Results of the factor analysis.

Item	Factor I	Factor II
The GP listened carefully to what I had to say	**0.808191**	0.082890
The GP understandably provided me with information about further diagnostic and treatment procedures	**0.718168**	0.148206
The GP gave comprehensive and clear answers to my questions	**0.843742**	0.028824
The GP was patient and attentive	**0.597365**	0.090705
The GP treated me with kindness and respect	**0.611880**	-0.152346
My GP supported me and understood my emotional needs	**0.717212**	0.330938
I believe the GP really cared about me and my health problems	**0.855058**	0.112973
I trust my GP and can give him even very personal information	**0.729440**	0.206707
I believe that teleconsultation helps to ensure the security and confidentiality of my data as a patient	0.165526	**0.702548**
I believe that teleconsultation saves time	0.066331	**0.584560**
I can easily use electronic medical records	0.017201	**0.657816**
I believe that the medical care I received during teleconsultation is as good as seeing my GP face-to-face	0.035106	**0.810745**
I felt comfortable discussing my health problems with my GP over the phone or on the Internet	0.128676	**0.747498**
I believe that teleconsultation is an acceptable way to solve my health problem	0.334380	**0.585482**

The reliability of both measurement scales was confirmed by the Cronbach’s alpha coefficient. The values of Cronbach’s alpha coefficient, showed a very good internal consistency of the GP- patient communication dimension (α = 0.88) and system experience dimension (α = 0.79). The results of the conducted reliability analysis of the measuring scale are presented in Table 4.

**Table 4:** Results of the reliability analysis of the measuring scales.

Factor/ Cronbach’s alpha coefficient	Item	Corr. item-Total corr.	Alpha if item delated
	The GP listened carefully to what I had to say	0.724	0.857
	The GP understandably provided me with information about further diagnostic and treatment procedures	0.644	0.859
	The GP gave comprehensive and clear answers to my questions	0.760	0.848
GP- patient communi-cationα = 0.884	The GP was patient and attentive	0.482	0.874
	The GP treated me with kindness and respect	0.462	0.876
	My GP supported me and understood my emotional needs	0.687	0.854
	I believe the GP really cared about me and my health problems	0.804	0.839
	I trust my GP and can give him even very personal information	0.676	0.860
	I believe that teleconsultation helps to ensure the security and confidentiality of my data as a patient	0.565	0.744
	I believe that teleconsultation saves time	0.441	0.769
The system experienceα = 0.786	I can easily use electronic medical records	0.482	0.763
	I believe that the medical care I received during teleconsultation is as good as seeing my GP F2F	0.654	0.714
	I felt comfortable discussing my health problems with my GP over the phone or on the Internet	0.623	0.722
	I believe that teleconsultation is an acceptable way to solve my health problem	0.455	0.768

The last part of the questioner concerned the demographic. The questions comprised the followings: age, gender, marital status, location, education, and professional activity. Finally, descriptive and simple summary statistics have been calculated, and multiple regression analysis has been used to explore if system experience impacts GP-patient communication quality. Data has been analysed using STATISTICA 13.1 (StatSoft) software program.

## Results

The respondents were mainly women (56%), and the mean age of the studied patients was 55 years. The highest percentage of responses came from Warsaw, where most of the clinics cooperating with the research team are located. As many as 5% of the respondents were unemployed; as a comparison, the national unemployment rate amounted to 6.2% in the period before the Covid-19 pandemic commenced. Hence, the majority of the respondents were represented by the employees (44.5%) and retirees or pensioners (44.5%). The largest group of respondents were the elderly in the group of over 65 years (39.4%). The smallest group were young people up to 24 years of age (5.1%). Out of 100 respondents to the survey, 45.5% declared that they make appointments with GP rarely (once a quarter), and 27.3% sporadically (once a year). Only 9.1% of patients make appointments very often—several times a month. The main method of contacting the GP was by phone. Only 5% of people used video consultation. The most common reasons for contacting GP were routine or non-urgent matters. A large proportion of the survey respondents (40.4%) reported that the main reason for the consultation were administrative matters: prescription, referral to a specialist, dismissal or a control (periodic) consultation (30.3%). Fewer respondents reported that they asked for a consultation because of preventive reasons (16.2%) and because of a first diagnosis or treatment (12.1%), which may be due to the fact that during a Covid -19 pandemic, patients avoid the consultation if they have not urgent reasons. Almost half of the respondents (46.5%) reported a very long waiting time for the expected teleconsultation (longer than 48 hours), which could have caused anxiety and lower the quality of medical teleconsultations. Proper communication is essential when the GP has not met the patient before and during telephone consultations, lacking typical visual cues. The overall satisfaction with communication within the teleconsultation was high, averaging 4.5, which was the best-noted dimension in assessing patient satisfaction of remote care. Patients rated the variables regarding empathy and respect as the bests, so they appreciated that the GP treated them with kindness and respect and were patient and careful. The phone calls are not conducive to the feeling of trust in the GP (Table 5).

**Table 5:** Variables used to measure the quality of communication with GP.

Variables	Mean	SD
The GP listened carefully to what I had to say	4.8	0.61
The GP was patient and attentive	4.8	0.58
The GP treated me with kindness and respect	4.8	0.53
The GP understandably provided me with information about further diagnostic and treatment procedures	4.4	1.07
The GP gave comprehensive and clear answers to my questions	4.6	0.81
The medical consultation time was sufficient	4.5	0.99
My GP supported me and understood my emotional needs	4.2	1.02
I believe the GP really cared about me and my health problems	4.2	1.11
I trust my GP and can give him even very personal information	4.1	1.26

Patients appreciate a quiet, unhurried teleconsultation where they feel heard and understood taking into account all their concerns. Demonstrating active listening was important because visual cues were not available to patients. Teleconsultation was well perceived by the surveyed patients, both in technical and behavioural aspects (Table 6), but our study didn’t confirm that a satisfaction with telemedicine is higher among women—mean for men = 3.95; mean for women = 3.85 [37, 38].

**Table 6:** Dimensions and variables used to measure the system experience.

Variables	Mean	SD
The teleconsultation went smoothly, without the technical problems	4.7	0.64
I believe that the technical support I received during the teleconsultation was adequate	4.4	0.99
I can easily use electronic medical records	3.1	1.69
I felt comfortable discussing my health issues with my GP over the phone or on the Internet	3.7	1.61
I believe that teleconsultation helps to ensure the security and confidentiality of my data as a patient	4.2	1.18
I believe that teleconsultation saves time	4.3	1.11
I believe that teleconsultation saves cost	3.8	1.35
I believe that the medical care I received during teleconsultation is as good as seeing my GP face-to-face	3.4	1.57
I believe that teleconsultation is an acceptable way to solve my health problem	3.4	1.63

Almost all patients (95%) believed that teleconsultation proceeded without technical disruptions, and according to 80% of respondents, the technical support received during teleconsultation was appropriate. However, this result is true mainly for phone calls because only a few patients in this study experienced a video consultation. Because “technical quality” for video consultation could be more problematic it should be assessed separately in the next study. The respondents did not notice technological barriers to teleconsultation in connectivity problems, poor internet access or mobile services. However, we shall note that teleconsultations are not suitable for patients with hearing impairments. Teleconsultation was quite convenient for patients, although they had never had experience with it before. Most of the respondents (62.6%) felt comfortable discussing their health problems with their GP over the phone or the Internet. It turned out that 55.5% of patients believe that the medical care they received during teleconsultation was as good as meeting their GP F2F. 33.3% of patients didn’t agree with this statement, and the remaining respondents did not have an opinion on this subject. The respondents pointed to the savings in time (83.8%) and money (65.7%) thanks to teleconsultation. On the other hand, many patients (37.4%) found it difficult to access electronic medical records. During the study, it turned out that teleconsultations were an acceptable way to solve 57.6% of the patient’s health problem.

The relationship between the quality of teleconsultation and communication was confirmed by the multiple regression analysis, which also examined the impact of various variables representing demographic data and the characteristics of teleconsultations on the quality of communication. The independent variables comprised: age, education, marital status, current work status, frequency of consultations, waiting time for a consultation, the reason for the consultation and experience with the system. The final selection of variables was performed using the "backward" stepwise regression analysis, which involves eliminating individual variables from the model until a satisfactory version is obtained. Standardized regression coefficients (beta) representing the impact of individual predictors on the explained variable defined by the quality of communication are presented in Table 7. This data regards a model that is characterized by the best fit and significance of all parameters (satisfactory p level).

**Table 7:** Multiple regression analysis results.

Variables	r	β value	t value	p-value
Age	0.21	0.06035	2.31085	0.000
Education	-0.21	-1.15456	-2.30446	0.0231
Waiting time for a consultation	-0.25	-1.32914	-2.91459	0.0235
Reason for the consultation	-0.32	-1.55253	-3.58270	0.0045
System experience	**0.38**	**0.32709**	**4.30936**	**0.0006**

The results of the conducted analysis indicate that the collectively employed independent variables allow for explaining 33.24% of the total variance of quality of GP- patient communication (R2 = 0.3324). The obtained by us results are statistically robust (Fisher’s F statistic = 8.9638; p <0.0005). The aforementioned results indicate that the four variables are statistically significant predictors of communication: age, education, waiting time for a consultation, the reason for the consultation and system experience. Factors negatively affecting the explained variable are: education waiting time for a consultation, the reason for the consultation. The research shows that the system experience has the most significant impact on the quality of communication.

## Discussion

Our results are aligned to the findings provided by other scholars, who postulate that, for a fraction of patients, the telemedicine not less, or perhaps more suitable than the personal care. This refers especially to those patients who face considerable barriers with access to GPs. Nonetheless, the telemedicine cannot replace the personal medical care in all cases. The telemedicine should not be used in sever or unstable conditions or whenever GPs’ examination is needed. Obtained by us results endorse the findings of other researchers [39–41], who claim some patients appreciate more F2F contact with a GP, primarily because of direct examination possibility, than the telemedicine. It appears to be especially important for the patients who have the choice and flexibility to use health services in the most appropriate way [19]. Despite concerns and reservations regarding teleconsultation, the majority of respondents, regardless of age and other characteristics, were willing to continue teleconsultation in the future. Our results endorse other studies [19] that, the convenience of telemedicine outweighs fears that patients cannot be physically seen or examined, in cases of routine health issue and when there had been already established relationship with a GP. Teleconsultation is best rated by those patients who are well versed in their health condition and when a consultation is about administrative matters [42, 43]. The high level of satisfaction with telemedicine may be due to lower expectations of GP at the time, especially when many patients felt that health services were less accessible. In fact, access to care improved for many of the patients who contacted GPs’ offices during the lockdown, due to less demand, which may have boosted positive feedback as well. Telemedicine was appreciated, primarily because the patients did not have to leave their premises and expose themselves to the potential infection.

Most of the patients in this study positively assessed the technical and behavioural quality of teleconsultation in primary care facilities during the Covid -19 pandemic. This happened despite the fact that telemedicine had not been used in Polish health care institutions before. Many patients appreciated the convenience and safety of telemedicine and the ability to be listened to without the risking infection. Our study has confirmed indications of other scientists, that that teleconsultations in general practices are rated well because of many advantages, including, inter alia, enabling patients to treat chronic diseases [44], convenience, and possibility to avoid potential infection [45].

The high ratings of the technical and the behavioural aspects of telemedicine contributed to high consideration of patients communication with GPs. Our findings regarding high assessment of communication during teleconsultation are supported by several studies that showed no differences in patients’ perceptions of the "interpersonal aspect of communication" during teleconsultation compared to F2F visits [46]. The earlier studies regarding GP- patient communication suggest that, despite physical separation, GP-patient communication during teleconsultation is not inferior to communication during F2F visits and it depends on technical and behavioural aspects of system experience [15, 47]. Moreover, the studies focused on specialist (oncology) care [48] showed that all surveyed patients were satisfied with the interpersonal aspects of teleconsultations, including GP-patient communication, and continued to use teleconsultation as part of their care. Conversely, other studies on partnership and rapport building found that teleconsultation reduces patient participation, and that GP are rarely involved in collaborative decision making and partnership building [49]. Our studies have also shown that the quality of the teleconsultations have a significant impact on the effectiveness of communication with a physicians. These results are aligned to other studies [50].

The main limitation of this study is the research sample, which comprise only four primary healthcare service providers in one country. This is a preliminary study however, of a pilot character, to be followed with the one on a larger scale. The relatively small size of our sample results primarily from the pandemic conditions, which has limited the research team interviewing possibilities. Anyway studying patient experiences and quality of communication during teleconsultation is essential for evaluating and improving services, especially when changes in service delivery are novel and unexpected [51]. Another limitation is due to the fact that remote care in Poland is mainly carried out through telephone consultations. Therefore, “Technical quality” needs to be assessed separately for phone calls and video consultation. At present video consultations in Poland are very rarely used so “technical quality” is not generalizable to other teleconsultation approaches than phone calls.

The study is also limited due to the fact that standards for teleconsultation have not yet been developed in primary health care in Poland. Subsequent studies should therefore take into account specialist outpatient care and focus on those specialties where such standards have been developed and in which a number of additional telemedicine tools can be used, such as systems for remote patient monitoring. It is worth emphasizing that special achievements in this area have recently been observed in neurology [52, 53].

Further improvement of the quality and access to telemedicine therefore requires the deployment of videoconferencing and systems for remote patient monitoring in specialist outpatient care. However, this would involve more support for patients who lack the technological capacity or the ability to use platforms and messaging [54]. More research is also needed on inequalities and the problems of differentiated access to technology. The presence of Internet access could be in the future a key determinant for the use of modern telemedicine. Thus, diffuse, affordable, and secure Internet access is required to develop Web-based interventions on the general population [55].

## Conclusion

Telemedicine might play a key role in controlling Covid-19 by enabling remote care for patients, who require the healthcare services, protecting them and the physicians from unnecessary virus exposure. Undertaken by us research has unfold the high potential of telemedicine consultations (mainly by telephone). Majority of the patients consider that teleconsultation is as good as F2F visit and acknowledge the positive effect on the quality of communication between the patient and the GP. During telephone consultations, the patients felt listened, understood, and experienced emotional support and respect from the GP. A particularly important feature of successful telemedicine has been the development of mutual trust in the clinician-patient relationship. Overall, patients reported a high level of satisfaction regarding communicating with their physicians during telecommunications. Teleconsultation was convenient and allowed patients to safely access healthcare services without having the fear of COVID-19 infection. Majority of patients felt comfortable in a great extent during medical consultations and acknowledged the security and confidentiality of their personal data. Communication with GP was best assessed by patients who needed diagnosis and consultations regarding treatment. On the other hand the system experience was better assessed by patients who asked for the routine consultations and administrative matters. Teleconsultation was less appropriate when a physical examination was necessary. Most patients find that teleconsultations are time and costs efficient, and acceptable way to address their health problems. We recommend to address better technological issues to enable more patients benefit from electronic health records. At present, most patients are not satisfied with this aspect. Nevertheless, offering teleconsultation as an option after limiting immediate access to GPs in times of a pandemic has the potential to increase quick and safe access to primary care for many patients.

The quality of communication during teleconsultation is mainly influenced by behavioural and technical aspects of the system experience. This is understandable as successful communication require good technical conditions, feeling of comfort, security and privacy and mutual trust between the patient and the GP. In our opinion, telemedicine, which was initiated during the lockdowns, should also be applied under normal conditions of healthcare provision, i.e. non-pandemic ones.

## Supporting information

S1 Data (XLSX)
